# Demasculinization of the *Anopheles gambiae* X chromosome

**DOI:** 10.1186/1471-2148-12-69

**Published:** 2012-05-18

**Authors:** Kalle Magnusson, Gareth J Lycett, Antonio M Mendes, Amy Lynd, Philippos-Aris Papathanos, Andrea Crisanti, Nikolai Windbichler

**Affiliations:** 1 Imperial College London, Department of Life Sciences, Imperial College Road, London, SW7 2AZ, UK; 2 Liverpool School of Tropical Medicine, Pembroke Place, London, L3 5QA, UK; 3 Division of Biology; California Institute of Technology, Pasadena, CA, USA

**Keywords:** *Anopheles gambiae*, demasculinization, germline x-chromosome inactivation, sexual antagonism, dosage compensation

## Abstract

**Background:**

In a number of organisms sex-biased genes are non-randomly distributed between autosomes and the shared sex chromosome X (or Z). Studies on *Anopheles gambiae* have produced conflicting results regarding the underrepresentation of male-biased genes on the X chromosome and it is unclear to what extent sexual antagonism, dosage compensation or X-inactivation in the male germline, the evolutionary forces that have been suggested to affect the chromosomal distribution of sex-biased genes, are operational in *Anopheles*.

**Results:**

We performed a meta-analysis of sex-biased gene expression in *Anopheles gambiae* which provides evidence for a general underrepresentation of male-biased genes on the X-chromosome that increased in significance with the observed degree of sex-bias. A phylogenomic comparison between *Drosophila melanogaster, Aedes aegypti* and *Culex quinquefasciatus* also indicates that the *Anopheles* X chromosome strongly disfavours the evolutionary conservation of male-biased expression and that novel male-biased genes are more likely to arise on autosomes. Finally, we demonstrate experimentally that transgenes situated on the *Anopheles gambiae* X chromosome are transcriptionally silenced in the male germline.

**Conclusion:**

The data presented here support the hypothesis that the observed demasculinization of the *Anopheles* X chromosome is driven by X-chromosome inactivation in the male germline and by sexual antagonism. The demasculinization appears to be the consequence of a loss of male-biased expression, rather than a failure in the establishment or the extinction of male-biased genes.

## Background

Several reports in model organisms have shown that sex-biased genes are not randomly distributed in the genome, which has led to speculation on the nature of the evolutionary forces that drive their chromosomal localisation. High-throughput gene expression studies have indicated that genes preferentially transcribed in the heterogametic sex of *DrosophilaCaenorhabditis elegansTribolium castaneum* and chicken are underrepresented on the X/Z chromosomes [1-5]. In *Drosophila melanogaster,* male-biased genes expressed in adult and pupal stages are underrepresented on the X chromosome [2,6]. In the adult male, the underrepresentation of X-linked genes occurs for genes expressed both in the testes and for some but not all somatic tissues [3,7]. The X chromosome of *C. elegans* is almost completely devoid of testes-expressed genes, but contains the expected number of somatic male-biased genes [4,8]. Contrary to *D. melanogaster* and *C. elegansMus musculus* early spermatogenic genes are overrepresented on the X chromosome [9], while those expressed later during meiosis are underrepresented on the X chromosome [10]. In *D. melanogaster* the genes transcribed in ovaries but not those expressed in other female tissues or in the soma are overrepresented on the X chromosome [3]. In other studies, female-biased *Drosophila* genes have been found to be both overrepresented [2] and randomly distributed [3,11] on the X chromosome, when overall expression in adults was considered. In *T. castaneum,* a significant overrepresentation of female-biased genes on the X chromosome was observed [1]. The variable patterns observed in these organisms may indicate different evolutionary pressures involved in the genomic distribution of sex-biased genes in these species.

At least three evolutionary forces have been suggested to explain the selective loss of male-biased genes on the X chromosome, namely X chromosome inactivation in the male germline, dosage compensation and sexual antagonism [12-15]. During male meiosis, the X chromosome of some organisms is thought to undergo condensation, thereby causing silencing of gene expression [12]. Although little is known about the molecular mechanism regulating germline X chromosome inactivation, its existence has been demonstrated in *C. elegans* and mammals [16,17] and is believed to contribute to the underrepresentation of X-linked genes expressed in meiosis in these species. Experimental evidence comes from studies in which the sperm-specific expression of transgenes located on *Drosophila* X chromosomes, was significantly lower than of those inserted into autosomes [18,19] although the occurrence of global meiotic X-inactivation in *Drosophila* has recently been called into question [20,21].

X chromosome inactivation cannot account for the non-random distribution of male-biased somatic genes observed in the *D. melanogaster* genome. Here dosage compensation and sexual antagonistic alleles, that are beneficial to one sex and harmful to the other, have been proposed as the mechanisms leading to the demasculinization of the *Drosophila* X chromosome [14,22]. Dosage compensation is a process that ensures equal levels of X-linked gene products for species in which the sexes differ in the number of X chromosomes. In a number of organisms, different mechanisms have been shown to balance expression of genes on the X chromosomes between hemizygous males and females, and also equalize expression between the X and autosomes [23]. Sex chromosome dosage compensation has been suggested to influence the genomic distribution of *D. melanogaster* male-biased genes. It has been suggested that the X chromosome would be an unfavourable location for genes to establish male bias, since they are already hyper-transcribed to achieve parity with autosomal genes [14]. This hypothesis is supported by evidence that correlates greater male bias with a lower likelihood that such a gene would be located on the X [14]. However, the existence of dosage compensation in the male *Drosophila* germline is still a controversial issue after many decades of study. Different statistical analysis of the same transcriptome data from normal *Drosophila* testes dissected into crude meiotic, as well as pre and post-meiotic stages has been interpreted as either demonstrating the presence [24] or absence [21] of dosage compensation in these tissues. Independent experiments on whole and dissected wild type testes [21] also appeared to support a lack of dosage compensation in the germline. However, because of the complications of heavily biased male expression in the testes, and an abundance of lowly expressed genes that are believed to skew the data, these results have been queried by a recent report that demonstrates dosage compensation throughout the male testes [25].

Sexual antagonism theory aims at explaining both the underrepresentation of somatic male-biased and overrepresentation of female-biased genes on the X chromosome. Since the alleles located on the X chromosome spend two-thirds of their time in the homogametic sex, such mutations will undergo significantly more selection in the female. Therefore, genes that are beneficial for the female are expected to accumulate and genes beneficial for the males are expected to move away from the X chromosome. However, this theory of sexual antagonism rests on the assumption that a significant part of these mutations is dominant [18], as recessive mutations beneficial to males would accumulate on the X chromosome. In *Drosophila*, male-biased genes exhibit a decrease, while female-biased genes exhibit an increase in X-linkage as the level of sex-bias increases [22]. Strongly sex-biased genes have been shown to represent dominant alleles in the fruit fly, and consequently the accumulation of highly female-biased genes and depletion of highly male-biased genes on the X chromosome is in support of sexual antagonism theory [22].

Regardless of the selection pressures that cause the dearth of male-biased genes, the proposed mechanisms by which the X chromosome could be demasculinized include a decreased rate of establishment of male-biased genes, an increased rate of male-biased gene loss, a reversal of male-biased gene expression, a decreased rate of genes evolving a male expression bias, relocation of X-linked male-biased genes, or a combination of these. *Drosophila* male-biased X-linked genes are poorly conserved in *Anopheles *[3], and are overrepresented among the *Drosophila* genes retroposed from the X chromosome to an autosome [13], thus suggesting that the X-linked male-biased genes show a high rate of gene loss and escape the X chromosome through retroposition. A decreased rate of establishment of novel male-biased genes on the *Drosophila pseudoobscura* neo-X chromosome has also been observed [11].

In contrast to other studied species, *A. gambiae* sex-biased genes have been described as randomly distributed [26,27]. However, recent studies found evidence of underrepresentation of testis-expressed genes on the *A. gambiae* X chromosome [28,29]. Furthermore, retrogenes were discovered to exhibit differences in the level of gene expression that depended on the location of the ancestral genes on either the autosomes or the X chromosome, suggesting a role for the suppression of X-linked gene expression in the male germline of *Anopheles *[29]. Baker and Russell also provided evidence that the male germline in *Anopheles* lacks dosage compensation, a fact that may also contribute to the deficiency of male-biased genes on the X chromosome [29].

Clearly further studies on X chromosome inactivation in the male germline, sexual antagonism or dosage compensation are needed in order to dissect the forces that are driving gene content evolution on the *Anopheles* X chromosome. In this study we performed an in-depth analysis of the genomic distribution of male- and female-biased *A. gambiae* genes by carrying out a meta-analysis across different sets of microarray expression data. We estimated, with high confidence, the magnitude of expression difference between sexes for large number of *A. gambiae* genes, and used this data to study the possible evolutionary forces that have directed the genomic localization of sex-biased genes. We also used transgenic approaches to experimentally assess the relative activity of testes-active regulatory regions when relocated to different positions on the autosomes and X-chromosome. Finally, we performed phylogenomic and codon bias analyses to understand the mechanisms that are likely to drive X chromosome demasculinization in *A. gambiae*.

## Results

### Chromosomal distribution of sex-biased genes

**Figure 1:**
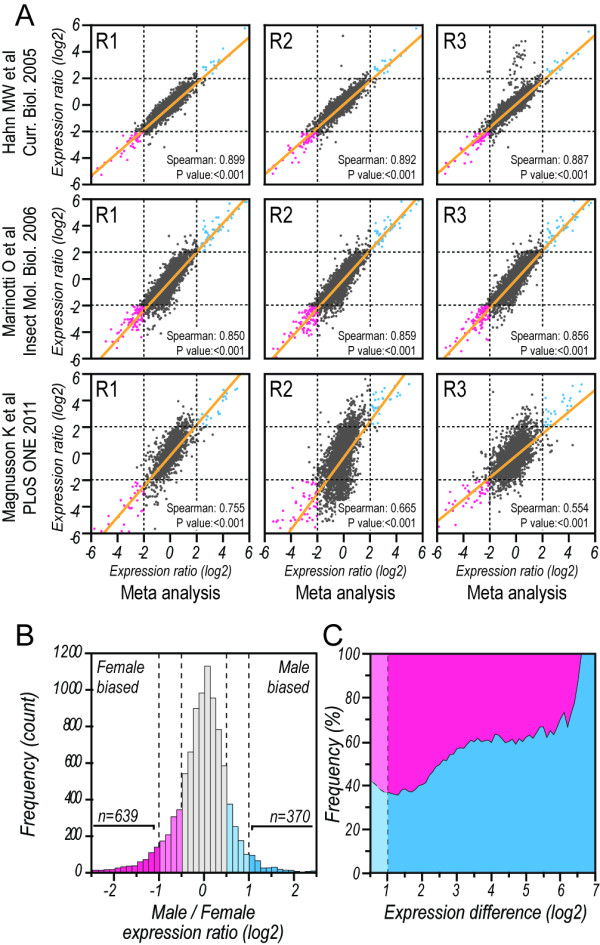
**Meta-analysis of genome-wide sex-biased expression in adult*A. gambiae.*A**) Meta analysis of male to female expression ratios and correlation with original expression data from published studies used for metadata computation. R1, R2 and R3 correspond to the biological replicates presented in each study. Each dot represents a gene, with genes presenting 2-fold increased expression in male *A. gambiae* when compared to females for both metadata and original reaction shown in blue, while genes presenting a 2-fold decrease for the same conditions are shown in pink. **B**) Histogram presenting the frequency of genes that were identified by the meta analysis for varying male to female expression ratios. **C**) Proportion of male- (blue) and female-biased genes (pink) and their expression difference in the meta analysis dataset.

**Figure 2:**
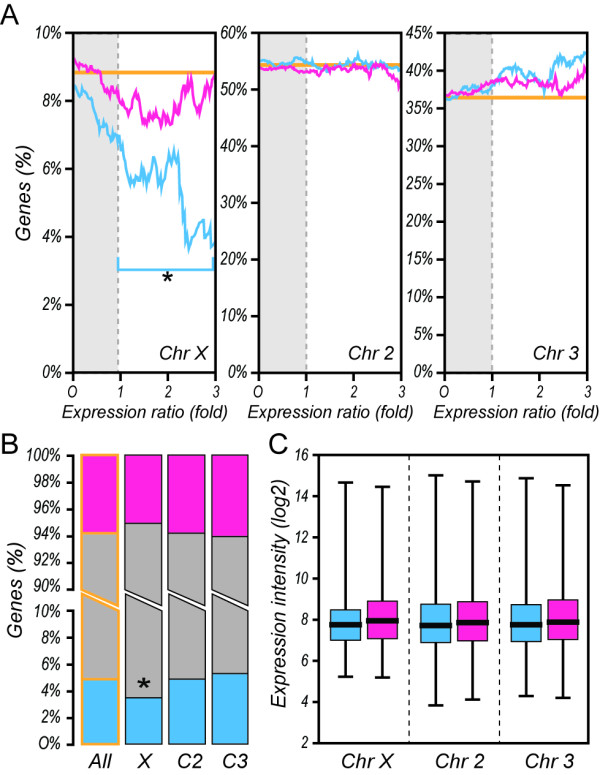
**Chromosomal linkage of sex-biased genes in adult*A. gambiae.*A)** Percentages of genes with increased expression in adult male (blue) or female (pink) *A. gambiae* mosquitoes are shown for the chromosomes X, 2 and 3 at increasing expression ratios. Significant differences in the number of genes with increased expression found in a particular chromosome were evaluated by hypergeometric distribution using as reference the overall number of genes linked to the same chromosome in the meta-analysis dataset (orange). **B**) Percentages of significantly regulated male (blue), female (pink) and non-biased genes (grey), as identified by RankProduct analysis, in the overall dataset, the X chromsome and the autosomes 2 and 3. Significant differences in the number of male and female biased genes observed were evaluated by hypergeometric distribution against the observed number for the same class in the overall dataset (*P* = <0.01:asterisk). **C**) Average chromosome-wide expression intensity per chromosome presented for male-biased genes and female-biased genes. Bar graphs presenting the average expression intensity for all genes in a particular chromosome (black line), + − 25th-75th quartile (box) and range of chromosome-wide hybridization intensities (whiskers) are shown.

We generated a meta-analysis dataset (Figure 1A) by applying a hierarchical Bayesian analysis framework to nine independent experimental replicates from three previously published microarray studies [26,30,31] that evaluated sex-biased gene expression levels in adult *A. gambiae*. This allowed us to identify 370 *A. gambiae* genes with a 2-fold or higher expression level in male mosquitoes in comparison to female mosquitoes, and 639 genes with this level of increase in expression in female mosquitoes (Figure 1B). Although genes with increased expression in females represent a higher proportion (>60 %) of the differentially regulated genes between sexes at the 2-fold cut-off, genes with increased expression in males are more numerous at higher ratios of expression bias (Figure 1C). To classify genes as male- or female-biased in a statistically stringent manner, we performed a Bayesian analysis on random permutations of the input data and applied a non-parametric statistical method to classify 467 genes as male-biased and 551 genes as female-biased, which were used for subsequent analysis (Additional file 1: Table S1). The distribution of genes amongst the male- and the female-biased fractions was then correlated with their chromosomal location (Figure 2). The female-biased genes do not deviate from the expected gene frequencies on any chromosome, whereas the X chromosome shows a significant reduction of male-biased genes (Figure 2B). The association between the degree of sex-bias and genomic location has been used as an argument for sexual antagonism in *Drosophila *[22]. We therefore examined whether increasing sex-bias was related to chromosomal distribution of adult-sex-biased *Anopheles* genes. We found that male-biased genes exhibit an inversely proportional relationship between the likelihood of location on the X chromosome and degree of sex-bias (Figure 2A). Interestingly, in contrast to *Drosophila,* the data does not indicate that the *A. gambiae* X-chromosome is a favourable location for highly female-biased genes. Global expression intensity of sex-biased genes did not vary between chromosomes (Figure 2C), thus indicating no inherent bias in the transcriptional activity of X-linked genes.

We also reanalyzed the microarray data from the mosquito study that had previously reported no significant reduction of adult male-biased genes on the X-chromosome [26]. This study was based on an early annotation of the *A. gambiae* genome, and when the original data was analyzed using contemporary annotation, we found a marginally significant underrepresentation of male-biased genes at a two-fold cut-off (*χ*^2^ = 4.056, *P* = 0.044) (−41.3 %) on the X chromosome. No other chromosome showed a significant over- or underrepresentation of male- or female-biased genes in this data. Sex-specific *A. gambiae* expression data has also recently become available for earlier developmental stages [31]. An analysis of the relatively low number of male-biased genes expressed during the immature stages shows a complete absence of X-linked genes at both the pupal (0 out of 65 male-biased genes) and 4^th^ instar larval (0 out of 61 male-biased genes) stages.

### Evidence for X linked inactivation in *Anopheles* testes

**Figure 3:**
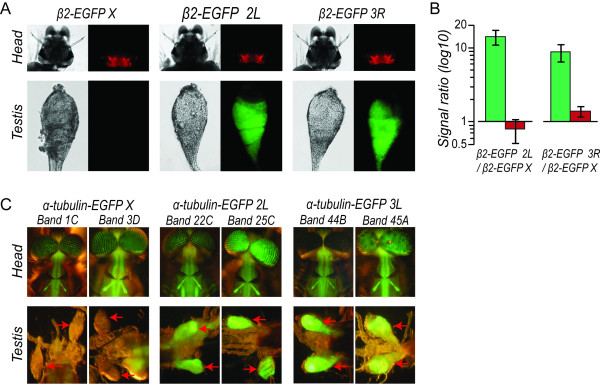
**Reporter gene expression of autosomal and X-linked transgenes in*A. gambiae.*** (**A**) Transmission and fluorescence (upper panels show RFP, lower panels show GFP) microphotographs of larvae heads and dissected testes taken from β2-eGFP 2 L, β2-eGFP-3R and β2-eGFP X mosquito lines. (**B**) Quantitative PCR analysis of the transcription activity of the neuronal 3xP3 and the testis specific β2-tubulin promoters. The bars show the ratios of quantitative RT-PCR signals detected in mosquito lines carrying autosomal integrations (β2-eGFP 2 L and β2-eGFP 3R) versus the X linked integration (β2-eGFP X) for the eGFP (green) and the DsRed (red) marker genes. (**C**) Expression activity of the α-tubulin promoter. Fluorescent microphotographs of six transgenic lines carrying single insertions of the α-tubulin-eGFP construct into either the X chromosome or one of the autosomes. The sites of chromosomal insertions are indicated on the top of the microphotographs. Red arrows in the photographs of the lower panel indicate testis.

We have also examined two sets of transgenic mosquitoes carrying alternative regulatory regions that direct somatic and testes-specific expression of fluorescent marker genes. In the first experiment, three transgenic lines (β2-eGFP 2L, β2-eGFP 3R and β2-eGFP X) were characterised that harboured a transformation construct carrying both eGFP, under the transcription control of a strictly testis-specific (β2 tubulin) promoter and DsRed, regulated by the eye-specific promoter 3xP3 [32,33]. In these lines, the transformation construct had inserted onto the *A. gambiae* chromosomes 2L, 3R and X. The 3 lines showed a comparable DsRed fluorescent intensity and in the two mosquito lines in which the transformation construct had integrated into the autosomes, we observed eGFP fluorescence localized in the testes (Figure 3A) starting from late 3^rd^ instar larvae and continuing throughout adulthood, faithfully reflecting the spatial and temporal transcription pattern of the β2-tubulin gene in developing sperm [32]. In contrast, we were unable to visually detect eGFP in vivo and in dissected testes in the β2-eGFP X mosquitoes (Figure 3A). Quantitative RT-PCR analysis indicated a 7 and 14 fold reduction in eGFP transcripts in testes from the β2-eGFP X line when compared with those from autosomal lines β2-eGFP 2L and β2-eGFP 3R respectively (Figure 3B). In comparison, no marked difference could be detected in DsRed transcription patterns of the three lines (Figure 3B).

To explore this further, we have also characterized a different set of transgenic *A. gambiae* lines (seven independent autosomal and two independent X-linked insertions) that contain a construct in which an α-tubulin promoter is driving eGFP expression. This promoter directs transgene expression in multiple somatic tissues, as well as the testes [34]. Significantly, in all lines carrying autosomal integrations of the reporter construct, we observed intense eGFP fluoresence in the spermogenic regions of the testes both by in vivo imaging and in dissected material (examples shown in Figure 3C). Testes specific expression was again observed from the third instar larval stage throughout further development. In both α-tubulin:eGFP lines carrying the construct inserted onto the X chromosome no eGFP signal was observed in vivo or ex vivo in the testis at any developmental stage under low magnification (adult testes - Figure 3C). Importantly, the core somatic expression pattern of eGFP did not vary between these transgenic lines (examples shown in Figure 3C). When expression was examined under higher magnification, fluorescence was observed in regions occupied by primary spermatocytes [35] and at greater intensity in regions carrying secondary spermatocytes, spermatids and mature spermatozoa in testes dissected from heterozygous α-tubulin:eGFP lines. No fluorescence was detectable in the apical hub region containing mitotic stem cells and their supporting somatic cells (Additional file 2: Figure S1). This distribution of expression has also been reported previously in the β-tub lines (Figure 3 and [33,36]). In contrast, after 10 fold longer exposure, only very weak eGFP expression was detected and only in the spermatid and spermatazoa regions in testes from hemizygous X linked lines (Additional file 2: Figure S 1).

Taken together, these experiments suggest that regulatory regions that are normally resident on the autosomes and actively drive expression in the testes, have greatly reduced activity in this tissue when they are relocated to the X chromosome. As such the X chromosome would be a particularly disfavoured location for genes to be expressed in the male germline.

### Gene birth, movement and loss of *Anopheles* X-linked genes

**Figure 4:**
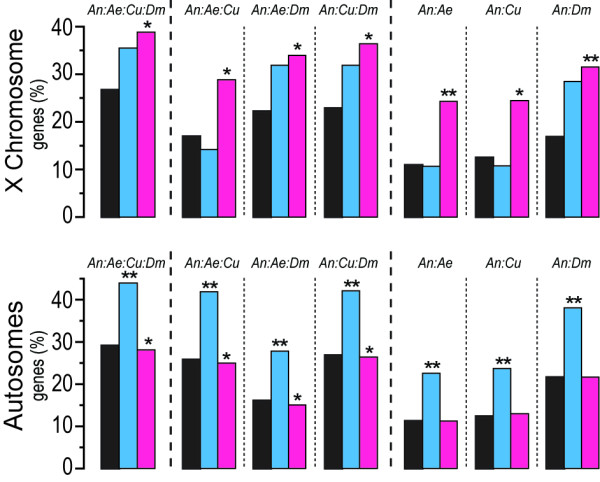
**Evolution of X-linked and autosomal sex-biased genes.** Percentages of male- (blue) and female-biased (pink) genes found to be unique when comparing the genomes of *A. gambiae* (An) with *A. aegypti* (Ae), *C. quinquefasciatus* (Cu) and *D. melanogaster* (Dm) are presented for X-linked (upper panels) and autosomal genes (lower panels). Statistically significant differences were evaluated by hypergeometric distribution using as reference the fraction of unique genes found in all X-linked or autosomal genes studied (*P* < 0.05, one asterisk; *P* < 0.01, two asterisks).

We assessed the rate of gene birth and extinction of *Anopheles* X-linked genes by calculating the frequency of *Anopheles*-specific (unique) genes and orthologous genes compared to the genomes of *Drosophila melanogaster, Aedes aegypti* and *Culex quinquefasciatus*. Using this approach, we found a significantly higher frequency of unique adult male-biased genes than non-sex-biased genes on the autosomes, while on the X chromosome, no significant increase or decrease in the frequency of unique male-biased genes was observed (Figure 4). In contrast, female-biased X-linked genes exhibit an increased number of unique genes, while a significantly lower frequency of adult female-biased unique genes was found on the autosomes (Figure 4). An opposite pattern for both male and female-biased genes was observed when studying the frequency of *D. melanogaster*, *A. aegypti* and *C. quinquefasciatus* orthologous genes (Additional file 3: Figure S2).

Furthermore, we compared the non-random use of synonymous codons for male and female biased X-linked genes, as well as autosomal genes, based on the assumption that genes that are rapidly evolving tend to have a low codon bias [37]. Using two metrics, the effective number of codons (ENC) and the frequency of optimal codons (F_OP_), we found that male-biased genes exhibit a significantly less biased codon usage than female-biased or non-biased genes on autosomes. This pattern of codon usage was not observed for X-linked male biased genes, suggesting that the evolutionary pressure which autosomal male-biased genes appear to be under is, at least in part, absent on the X chromosome (Additional file 4: Table S2). Such difference in the levels of codon usage do not seem to be caused by differences in gene length, as autosomal male-biased genes are on average smaller than non-biased and female-biased autosomal genes (Additional file 4: Table S2). This analysis also indicates that the evolutionary pressure for conservation is generally higher in X-linked genes than autosomal genes, independently of sex bias expression, as had previously been suggested for *D. melanogaster* and *C. elegans *[38].)

Retroposition of male-biased genes off the X chromosome, a factor in the demasculinization of the *Drosophila* X chromosome [13], also appears to be a mechanism acting in the mosquito [29]. A recent study found a high degree of retrogene movement off the *Anopheles* X-chromosome after the evolutionary split with *A. aegypti,* but no excess retrogene movement was observed since the split with *D. melanogaster *[39]. We find that genes in our high confidence dataset that represent paralogs are overrepresented to a greater extent within the set of X male-biased genes than in the set of autosomal male-biased genes (Additional file 5: Figure S3). This finding indicates that male-biased genes on the X give rise to a higher number of paralogs, and suggests that on average, not only are more such genes duplicating, but they also duplicate more often.

### Transcriptional evolution of sex-biased genes

**Figure 5:**
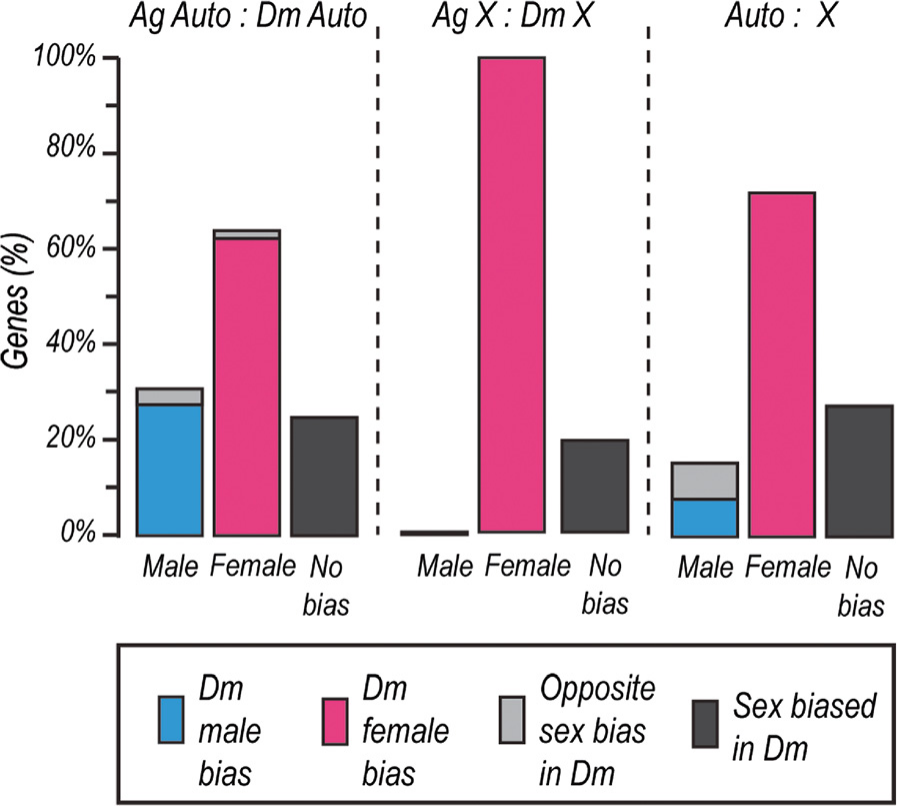
**Conservation of sex-biased expression of X-linked and autosomal orthologs in*A. gambiae *and*D. melanogaster.*** The transcription profile of male-, female and non sex-biased *A. gambiae* genes was compared to their one-to-one *D. melanogaster* orthologs. The bars show the proportion of *A. gambiae* sex biased genes with a *D. melanogaster* ortholog showing either a conserved male (blue), female (pink) or a reversed (light gray) transcription pattern. The proportion of *A. gambiae* non sex-biased genes with sex-biased *D. melanogaster* orthologs is also shown (dark grey). Shown is the percentage of such genes according to their location in both *A. gambiae* (Ag) and *D. melanogaster* (Dm) on either the autosomes (Auto) or the X chromosome (X).

The effect of chromosomal location on the conservation of sex-biased expression was studied in a comparative analysis of *A. gambiae* and *D. melanogaster* one-to-one orthologous genes present in our meta-analysis dataset (Figure 5). Both the male- and female-biased orthologs that are located on autosomes in both species show a high degree of conservation of sex-biased expression. Mosquito female-biased genes that are autosomal in one species and X-linked in the other species show a high degree of conservation of their sex-biased expression. All of the *Anopheles* X-linked female-biased genes that have *D. melanogaster* orthologues with conserved X-chromosomal location maintain their sex-biased expression in *Drosophila*. Similarly, approximately half of *Anopheles* male-biased genes which are X-linked in one species and autosomal in the other species maintain their male-bias. Strikingly, no genes that are male-biased and X-linked in both *Anopheles* and *Drosophila* could be identified.

## Discussion

In *DrosophilaC. elegans* and *M. musculus* there is evidence for complex selective pressures driving the non-random chromosomal distribution of sex-biased genes. *Anopheles* was long thought to share the same ancestral X chromosome with *Drosophila*. Thus X-inactivation in the male germline, sex chromosome dosage compensation and sexually antagonistic evolution believed to be acting on the fruit fly X chromosome were similarly thought to operate on the *Anopheles* X. New evidence suggests that the *Anopheles* and *Drosophila* X chromosomes were formed independently from the same pair of autosomes [39]. In addition, *A. gambiae* sex-biased genes, unlike *Drosophila*, had been regarded to have a random chromosomal distribution due to a previous report that failed to detect any significant deviation in the distribution of male-biased genes in *A. gambiae *[26,27]. Together these recent findings suggested significant differences in the evolutionary forces that shaped the mosquito and fruit fly X chromosomes.

Combining data from three different *A. gambiae* microarray studies we compared adult male and female gene expression and found that adult male-biased genes are in fact significantly underrepresented on the X chromosome. *Drosophila* studies regarding the chromosomal distribution of female-biased genes illustrate that differences in data-analysis and microarray platforms can occasionally generate disparate results [2,3,11]. However, our reanalysis of the Hahn and Lanzaro microarray study with contemporary genome annotation indicates that this data already exhibits a tendency for X chromosome demasculinization [26].

We also provide independent sets of experiments showing that the testis-specific β2-tubulin and the more widely active α-tubulin promoters are notably less active in the male gonads when the transgenes had an X-linked location compared to an autosomal one. In *Drosophila* spermatogenesis, it is thought that global transcription is shut down upon onset of meiosis, and stored transcripts are translated, as required, during later stages. Recent evidence would also suggest the transcription of certain genes in post-meiotic stages in *Drosophila *[40] and *An. gambiae *[35]*.* Our data demonstrates loss of GFP fluorescence in primary spermatocytes and supports the role of premature X inactivation during these meiotic stages in the testes.

This expression pattern is supported by in situ analysis in Aedes mosquitoes indicating that β2 tubulin transcription is initiated in the primary spermatocytes [41]. However, it has been suggested [21] that X linked silencing of transgenes is established in pre-meiosis by an unknown mechanism. We thus cannot entirely rule out that transcription occurs in earlier stages and the visible products of translation only become apparent in the primary spermatocytes. Nevertheless, our findings establish the presence of X chromosome inactivation in the *Anopheles* male germline and the inference that localization on the X chromosome would interfere with the regulation of male-specific genes during the process of spermatogenesis.

Importantly, although we have examined only a limited number of transgenic lines, the results support the conclusions from related experiments in *Drosophila *[18,19] that had been cast into doubt by recent studies that argued against global X-inactivation in *Drosophila *[20,21] and dosage compensation in the germline [21]. The results of these latter studies could be partially explained by the general paucity of testis-specific genes on the X chromosome [3,11] and the skewed gene content [25]. A recent report [25] using mutants enriched in mitotic cells offers evidence of dosage compensation throughout the male testes.

Little is known about somatic and germline dosage compensation *Anopheles* and differences in this mechanism from *Drosophila* could account for differences in X-linked expression of sex-biased genes. In our transgenic analysis, the level of somatic male expression from the X-linked α-tubulin promoter appears to lie between that observed in hemizygous and homozygous females (Additional file 6: Figure S4). Likewise from microarray analysis, Baker and Russell found evidence supporting the hypertranscription of the X chromosome in the male soma, but observed that the X-linked germline-expressed genes were only expressed at about half the intensity compared to autosomal genes; perhaps indicating that the X chromosome is not hypertranscribed in the *A. gambiae* male germline [29]. However, in the absence of mutant strains, similar to those used in *Drosophila *[25], it is difficult to quantify dosage compensation in the germline because of extensive sex-specific expression in these tissues, and the confounding consequences of germline X-inactivation. In addition, further data is needed to determine whether alternative regions of the *Anopheles* X chromosome are subject to differential dosage compensation and whether transgenic insertions are regulated correctly by dosage compensation mechanisms. The hypothesis that a lack of dosage compensation in the germline is the cause for reduced transgene expression is difficult to reconcile with the barely detectable GFP signal in the X linked lines, and qPCR data suggesting a 7 to 14 fold higher transcription in whole testes in the heterozygous autosomal lines. If transcriptional dosage compensation was strictly linear, in its presence, more expression would be expected in the X linked lines, whereas a similar level of transcription would be observed in the absence of dosage compensation.

If, however, dosage compensation doesn’t occur in the male germline of *Drosophila* and also *Anopheles* then the selective drive for male biased genes away from the X chromosome would be even greater, and would contribute to their underrepresentation.

The observed X-linked silencing of expression may thus be causally related to the demasculinization of the *A. gambiae* X chromosome in relation to testes-specific genes, but the observed underrepresentation of *A. gambiae* (and *Drosophila*) somatic male-biased X-linked genes suggests that other mechanisms also contribute to the dearth of male-biased genes on the X chromosome [3,28]. The complete absence of male-biased X-linked genes during the pre-adult stages equally suggests that the demasculinization of the *Anopheles* X chromosome may be driven by evolutionary forces acting throughout multiple life stages and that are not confined to specific tissues such as the testes.

A mechanism that affects all X-linked genes is sexual antagonism. Sexual antagonism theory predicts that since the X-linked alleles undergo proportionally more selection in females, the genes that are beneficial for females are expected to accumulate and genes beneficial for males are expected to be lacking on the X chromosome. This assertion only holds if the majority of the mutations are dominant and in *Drosophila* a link has been established between a stronger sex-bias and dominance [22]. Our observation of a negative correlation between the degree of *A. gambiae* adult male-bias and X-linkage is consistent with sexual antagonism theory. However unlike *Drosophila,* the mosquito female-biased genes do not show an increase in X-linkage at an increasing level of sex-bias, thus highlighting potential differences in evolutionary forces acting upon X-linked female-biased genes between *Anopheles* and *Drosophila*.

If the inactivation of the X chromosome in the male germline and sexual conflict drive demasculinization what is the mechanism by which the X chromosome sheds its male genes? A decreased rate of establishment of novel X linked male-biased genes has been suggested as an important contributor to the demasculinization of the X chromosome in *Drosophila *[27]. We show, by comparing *Anopheles* to other species of mosquitoes and *Drosophila*, that *Anopheles* female-biased genes display a very high degree of evolutionary conservation on the autosomes, but not on the X chromosome. In contrast, autosomal male-biased genes show a significantly higher degree of gene birth and extinction, yet no such pattern was observed within the set of X-linked male genes. Additionally, autosomal but not X-linked male-biased genes exhibit a higher rate of sequence divergence, as indicated by codon bias analysis. The use of a higher number of effective codons and a decreased preference for optimal codons is indicative of an accelerated rate of evolution of these genes. A high rate of gene birth and extinction of *Anopheles* male-biased genes has been previously observed, but here we observe that this pattern appears to be restricted to autosomes [31]. When comparing sex-biased expression conservation with respect to chromosomal location in *A. gambiae* and *D. melanogaster,* a correlation is observed between chromosomal location and the conservation of male-biased expression, in which X-linkage is associated with a high degree of loss of male-bias. The *A. gambiae* female-biased genes exhibit a high degree of expression conservation in *D. melanogaster* irrespective of their chromosomal location, implying that, unlike the situation in the male, X-linkage is not associated with an increased expression divergence in the female. We conclude that the demasculinization of the *Anopheles* X chromosome is not due to a strongly decreased rate of establishment of novel male-biased genes, or to a high rate of gene extinction, but rather due to the loss of male-biased expression of such genes.

Complex evolutionary forces are at work when it comes to the X chromosome and the expression of sex-biased genes. Here we significantly further our understanding of the chromosomal distribution of sex-biased genes and the contributing causes and mechanisms by which the X chromosome is demasculinized. Next generation sequencing approaches in a number of different mosquito species may help to further elucidate the factors driving sex-biased gene expression and its relationship to X-linkage.

## Materials and Methods

### Meta-Analysis

Available microarray datasets on male and female *A. gambiae* sex biased expression [26,42] were downloaded from the GEO data repository of NCBI or used as provided in the original publication [31]. Affymetrix microarray data [26,42] was normalized using the gcRMA library of the R integration for the BioProspector package, excluding probes with intensities less than 50. Male/female expression ratios were calculated for the various experiments included in the three datasets and used as input for BAGEL [43]. This program was used to infer relative gene expression levels between male and female adult *A. gambiae* mosquitoes and their 95 % confidence interval. To classify genes as male or female biased genes, a P value for significant differential regulation between sexes and an estimated percentage of false positive predictions (PFP) was calculated by applying the RankSum method of the RankProd biocondutor package [44] to results from re-running the BAGEL program on random permutations of the input data (PFP cut-off ≤ 0.001 and *P* value cut-off ≤ 0.0001). Overall, adult male/female expression ratios were determined for 9447 *A. gambiae* genes, 467 of which present significantly increased expression in male mosquitoes, while 551 genes present a significant increase on expression in female mosquitoes (Additional file 1: TableS1). Additional analysis revealed that 370 genes presented a 2-fold increase of expression in male mosquitoes and 639 genes presented identical increase in female mosquitoes (Figure 1).

### Co-localization, comparative genomics and transcriptomics

The AgamP3.6 assembly of Vectorbase (December 2010) was used to obtain information on the chromosomal location, parology, orthology and sequence data for all *Ae. AegyptiC. quinquefasciatusD. melanogaster* and *A. gambiae* genes analyzed [45]. Statistically significant differences in the chromosomal distribution of sex-biased genes on the X chromosomes, as well as changes in the frequency of unique genes and orthologs were detected by applying a hypergeometric distribution and calculated with Excel (Microsoft Corp., Redlands, WA).

For codon usage analysis, a collection of 8040 sequences was assembled after applying several sequence quality control steps as previously suggested [46], including: presence of an ATG start codon, sequence length being a multiple of three, absence of internal stop codons and selection of only the longest transcript in the case of genes with multiple transcripts. CodonW (http://bioweb.pasteur.fr/seqanal/interfaces/codonw.html) was used to calculate the effective number of codons (ENC) [47] and the frequency of optimal codons (F_OP_) [48]. Statistical differences from the values obtained for all X-linked and autosomal genes evaluated were evaluated by applying the nonparametric Mann–Whitney test (GraphPad Prism V4). According to the ENC index, lower values than the reference set indicate stronger codon usage bias which is associated to slower evolutionary rates while higher values indicate weaker codon usage bias and selection intensity. The opposite association is expected from the F_OP_ index.

Data of *Drosophila melanogaster* sex-biased expression was obtained from the Sebida database (http://141.61.102.16:8080/sebida/index.php) based on a meta-analysis of previously published data [3] and a non redundant set of one to one orthologs with *A. gambiae* genes was established for specifically this analysis (Additional file 7 Table S3).

### Development of Transgenic Lines

The transgenic lines β2-eGFP 2 L, β2-eGFP 3R and β2-eGFP X were developed using the plasmid pPB{3xP3-DsRed}β2-eGFP::I-PpoI as previously described [33]. Briefly, *A. gambiae* (strain G3) embryos were injected using a Femtojet Express and sterile Femtotips (Eppendorf) with 0.2 μg/μl of plasmid and 0.8 μg/μl of piggyback helper RNA. The injected individuals were raised and crossed as adults to wild type mosquitoes. The progeny (G1) of these crosses were screened for 3xP3 DsRed fluorescence and independent lines established. The β2-eGFP construct integrated at position 4549156 (ATCATATTCATTA piggy-bac TTAATATGTACGAC) on the X chromosome as determined by inverse PCR in the line β2-eGFP X. The integration of the same construct on two distinct autosomal locations (line β2-eGFP 2 L and line β2-eGFP 3R) was previously reported [33] and heterozygote mosquitoes were used for all experiments. Digital images of larvae and dissected tissues were captured using a Nikon Eclipse TE200 and a Nikon DXM1200 digital camera. The pBDsRed_atubgfp transgenic lines were produced as described [34]. Briefly, G3 embryos were injected using a FemtoJet Microinjector and quartz needles (Sutter) with a 3.5:1.5 ratio of pBDsRed_atubgfp:phspBac [49] plasmids at a total concentration of 500 ng/μl. G1 progeny were screened for DsRed and eGFP expression and independent lines established. Chromosome insertion sites of the different lines were determined by inverse PCR, of which one was located on the X chromosome and seven were autosomal. All of these integration sites and flanking sequences have been described recently [34]. In addition, a previously unreported X linked insertion site (5131028 CTGAAAAGAATTAA-piggyBac-TTAAAACAGTTAAT) was identified within a mixed family line carrying multiple integrations of the transgene (Line E). To purify the X linked transgene, sequential backcrosses to the wild type strain were performed. Female progeny from isofemale lines carrying the X linked transgene (identified by PCR) were crossed to wild type males and their resultant progeny scored for eGFP. Four backcrosses were required to produce a line that gave inheritance consistent with a single copy transgene. Males from this line were then backcrossed to wild females to verify X linked inheritance (female only transgenic progeny), and then kept by inbreeding and enrichment of the transgenics. Images of pBDsRed_atubgfp pupa, adults and dissected tissues were captured using a Leica MZFLIII fitted with a Nikon P5100 digital camera, or an Olympus BX60 epifluoresence microscope fitted with a Nikon DSU2 camera.

### Authors’ contributions

KM, GJL, AMM, AC and NW contributed to the conceptual development of the work and the writing of the manuscript. KM, GJL, AL, PAP and NW carried out the experiments. KM and AMM performed data analyses. All authors read and approved the final version of the manuscript.

## Supplementary Material

Additional file 1 **Table S1.** Subset of genes derived from the meta-analysis dataset that were classified as male- or female-biased in a statistically stringent manner. Click here for file

Additional file 2 **Figure S1.** Reporter gene expression in pupal testes dissected from *A. gambiae* transgenic lines that are heterozygous (autosomal) and hemizygous (X-linked) for the α-tubeGFP transgene. Transmission (left), fluorescence (middle) and merged (right) microphotographs showing the extent of eGFP expression in testes dissected from heterozygous α-tubulin-EGFP 3 L (top row) and hemizygous α-tubulin-EGFP X lines. Letters denote regions of the testes containing the following spermatogenic stages as described in [35]: H – hub, P - .Primary Spermatocytes, S – Secondary Spermatocytes, Sd – Spermatids, and Sp – Spermatozoa; Scale bar, 50 μm. Fluorescent images were taken with identical camera settings, except to allow visualization of very weak fluorescence in testes from the X linked lines, the exposure time was increased 10 fold to 100 ms (at 10 ms no fluorescence was observed). Click here for file

Additional file 3 **Figure S2.** Evolution of X-linked and autosomal sex-biased orthologs. Percentages of male- (blue) and female-biased (pink) orthologous genes identified when comparing the genomes of *A. gambiae* (An) with *A. aegypti* (Ae), *C. quinquefasciatus* (Cu) and *D. melanogaster* (Dm) are presented for X-linked (upper panels) and autosomal genes (lower panels). Statistically significant differences were evaluated by hypergeometric distribution using as reference the fraction of orthologous genes found in all X-linked or autosomal genes studied (*P* < 0.05, one asterisk; *P* < 0.01, two asterisks). Click here for file

Additional file 4 **Table S2.** Levels of codon bias for X-linked and autosomal sex-biased genes in *A. gambiae*. N indicates number of genes; Length indicates the mean number of base pair nucleotides for each category; ENC indicates the mean effective number of codons for each category; Fop indicates the mean frequency of optimal codons for each category. P-value for Mann-Whitney two-sample tests comparing values for male or female biased genes to the total number of genes in each category are present within brackets. Click here for file

Additional file 5 **Figure S3.** Duplication events in sex-biased genes of adult *A. gambiae* mosquitoes. A) Percentages of male- (blue) and female- biased (pink) genes located on the X chromosome or the autosomes of *A. gambiae* mosquitoes that were separated by a duplication event and present at least one paralogous sequence. Statistically significant differences were evaluated by hypergeometric distribution using as reference (grey) the percentages of genes found within the set of all X-linked or autosomal genes studied (*P* < 0.05, one asterisk; *P* < 0.01, two asterisks) B) Average number of duplication events and paralog sequences presented by male and female-biased genes. Statistical differences were evaluated by Mann–Whitney test against the observed events in all genes studies on either the X chromosome or in the autosomes. Click here for file

Additional file 6 **Figure S4.** Reporter gene expression of autosomal and X-linked transgenes in homozygous and hemizygous *A. gambiae.* Transmission (upper panels) and fluorescent microphotographs (GFP middle panels, RFP lower panels) showing the expression activity of male (M) and female (F) pupae of the α-tubulin promoter α-tubulin-EGFP X, α-tubulin-EGFP 2 L and α-tubulin-EGFP 3R transgenic lines carrying either one or two copies of the α-tubulin-eGFP construct into either the X chromosome or on chromosomes 2 L/3R. Click here for file

Additional file 7 **Table S3.** Non redundant set of one to one orthologs between *A. gambiae* and *D. melanogaster* that was used as a basis for the codon bias analysis. Click here for file
