# Supplementary figures and images for: Demasculinization of the Anopheles gambiae X chromosome
Source: BMC Evol Biol. 2012 May 18;12:69. doi: 10.1186/1471-2148-12-69 (PMC3428665; doi:10.1186/1471-2148-12-69)

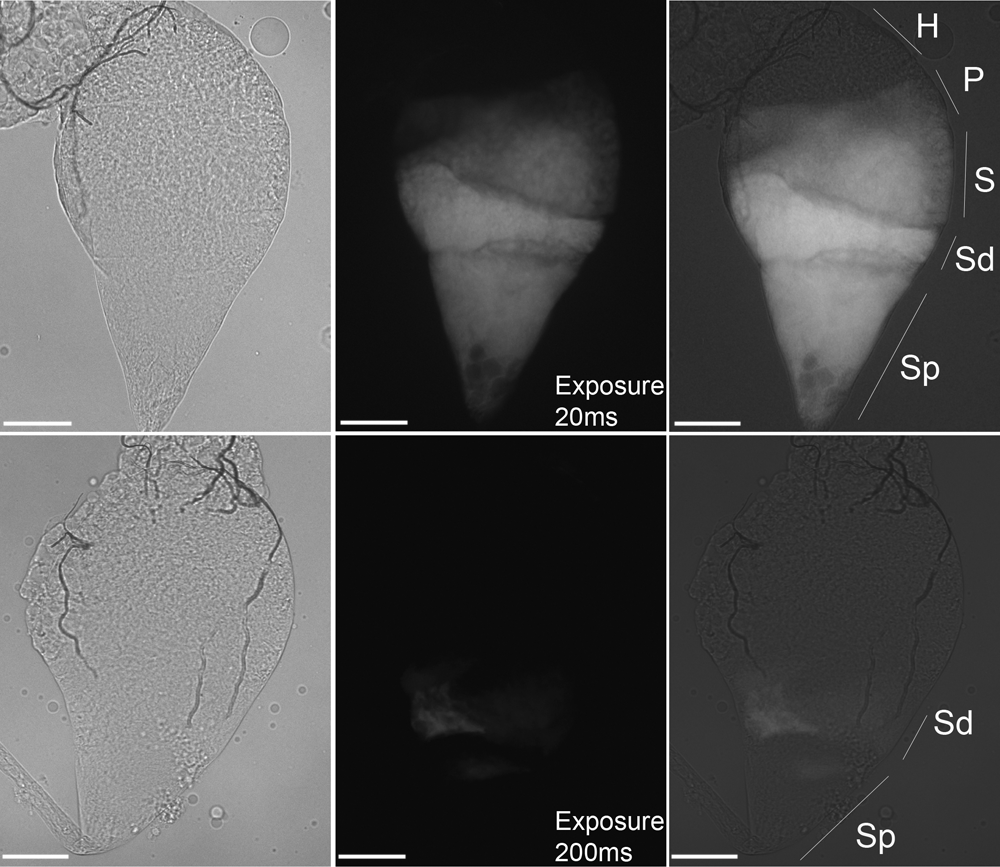

Supplement: Additional file 1 — Table S1. Subset of genes derived from the meta-analysis dataset that were classified as male- or female-biased in a statistically stringent manner. [file 1471-2148-12-69-S1.tiff]

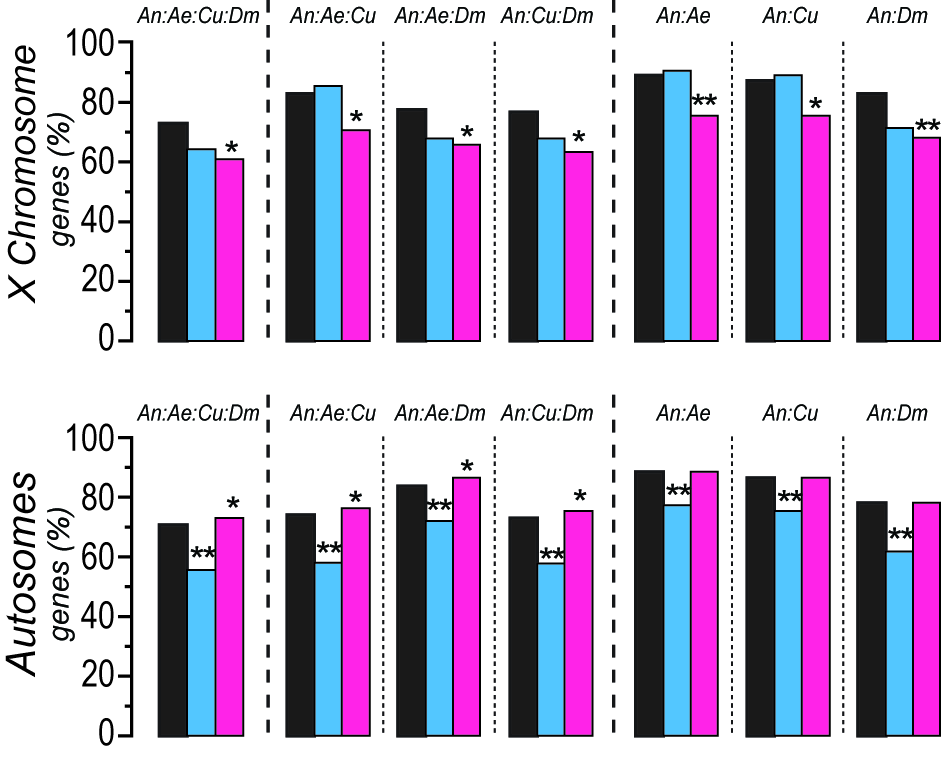

Supplement: Additional file 3 — Figure S2. Evolution of X-linked and autosomal sex-biased orthologs. Percentages of male- (blue) and female-biased (pink) orthologous genes identified when comparing the genomes of A. gambiae (An) with A. aegypti (Ae), C. quinquefasciatus (Cu) and D. melanogaster (Dm) are presented for X-linked (upper panels) and autosomal genes (lower panels). Statistically significant differences were evaluated by hypergeometric distribution using as reference the fraction of orthologous genes found in all X-linked or autosomal genes studied (P < 0.05, one asterisk; P < 0.01, two asterisks). [file 1471-2148-12-69-S3.tiff]

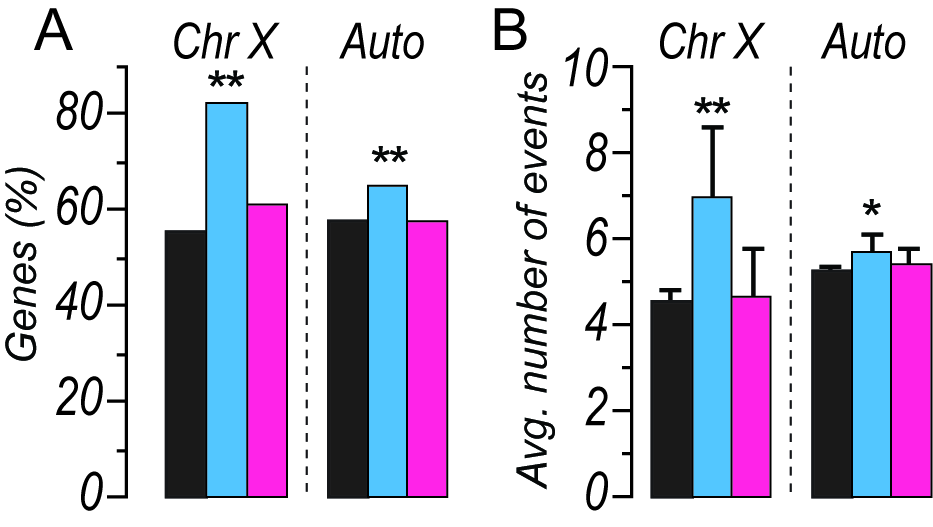

Supplement: Additional file 5 — Figure S3. Duplication events in sex-biased genes of adult A. gambiae mosquitoes. A) Percentages of male- (blue) and female- biased (pink) genes located on the X chromosome or the autosomes of A. gambiae mosquitoes that were separated by a duplication event and present at least one paralogous sequence. Statistically significant differences were evaluated by hypergeometric distribution using as reference (grey) the percentages of genes found within the set of all X-linked or autosomal genes studied (P < 0.05, one asterisk; P < 0.01, two asterisks) B) Average number of duplication events and paralog sequences presented by male and female-biased genes. Statistical differences were evaluated by Mann–Whitney test against the observed events in all genes studies on either the X chromosome or in the autosomes. [file 1471-2148-12-69-S5.tiff]

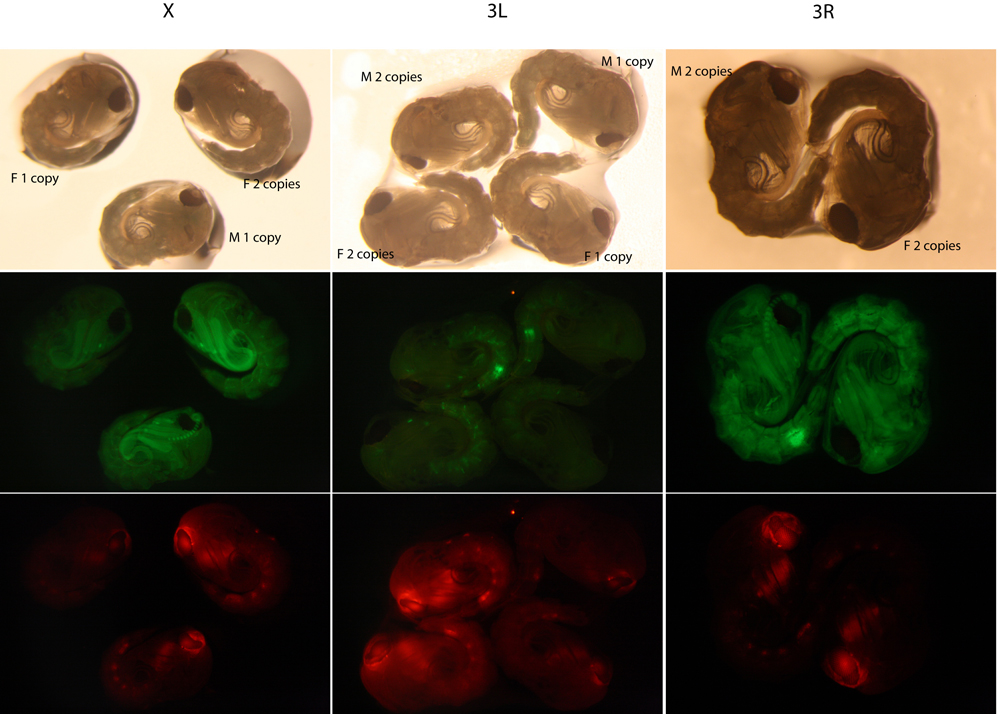

Supplement: Additional file 7 — Table S3. Non redundant set of one to one orthologs between A. gambiae and D. melanogaster that was used as a basis for the codon bias analysis. [file 1471-2148-12-69-S7.png]
