# Supplementary material for: Demasculinization of the Anopheles gambiae X chromosome
Source: BMC Evol Biol. 2012 May 18;12:69. doi: 10.1186/1471-2148-12-69 (PMC3428665; doi:10.1186/1471-2148-12-69)
Supplement: Additional file 4 — Table S2. Levels of codon bias for X-linked and autosomal sex-biased genes in A. gambiae. N indicates number of genes; Length indicates the mean number of base pair nucleotides for each category; ENC indicates the mean effective number of codons for each category; Fop indicates the mean frequency of optimal codons for each category. P-value for Mann-Whitney two-sample tests comparing values for male or female biased genes to the total number of genes in each category are present within brackets. [file 1471-2148-12-69-S4.doc]

|  |  | **N total** | **Length** | **ENC** | **F_OP_** |
| --- | --- | --- | --- | --- | --- |
|  |  |  |  |  |  |
|  |  |  |  |  |  |
| **X**  **Chromosome** | Total | 740 | 612 | 38.38 | 0.79 |
|  |  |  |  |  |  |
|  | Male biased | 20 | 496 (P=0.941) | 38.03 (P=0.831) | 0.81 (P=0.708) |
|  |  |  |  |  |  |
|  | Female biased | 36 | 436 (P=0.121) | 38.30 (P=0.754) | 0.79 (P=0.524) |
|  |  |  |  |  |  |
|  |  |  |  |  |  |
| **Autosomes** | Total | 7118 | 549 | 45.86 | 0.67 |
|  |  |  |  |  |  |
|  | Male biased | 356 | 408 (P<0.001) | 47.53 (P<0.001) | 0.63 (P<0.001) |
|  |  |  |  |  |  |
|  | Female biased | 440 | 445 (P<0.001) | 45.57 (P=0.593) | 0.66 (P=0.128) |
|  |  |  |  |  |  |

**Levels of codon bias for X-linked and autosomal sex-biased genes in *A. gambiae*.**

N indicates number of genes; Length indicates the mean number of base pair nucleotides for each category; ENC indicates the mean effective number of codons for each category; Fop indicates the mean frequency of optimal codons for each category. P-value for Mann-Whitney two-sample tests comparing values for male or female biased genes to the total number of genes in each category are present within brackets.
